# Biomarkers in colorectal cancer screening

**DOI:** 10.18632/oncotarget.22991

**Published:** 2017-12-06

**Authors:** Katarzyna Schab, Aleksandra Tańska, Maciej Smutek

**Affiliations:** Maciej Smutek: Clinical Laboratory, St. John of Dukla Cancer Center, Lublin, Lubelskie, Poland

**Keywords:** colorectal cancer, screening, high-throughput sequencing, personalised medicine, biomarkers

CRC or Colorectal Cancer is known to be one of the most prevalent and lethal neoplasms. Every individual of age 50 and above should undergo a regular screening. Nowadays, depending on the region, recommended preventive procedures are faecal occult blood testing followed by subsequent colonoscopy, or regular colonoscopy alone.

Intestinal endoscopy is one of the best methods of adenomatous polyps detection. Notwithstanding this fact, the procedure is invasive and might cause unpleasant or dangerous side effects [1, 2].

On the other hand, faecal occult blood testing is relatively inexpensive. Moreover, the sample is easy to gather. However, attention should be paid to false results distorting the diagnosis. We deal more often with false positive results due to high prevalence of haemorrhoids in the adult population. It raises an important point that faecal occult blood testing may increase the incidence of unnecessary colonoscopy. On the other hand, false negative results constitute a major barrier to early detection of CRC and thus the success of screening. Several adenomatous polyps do not cause bleeding at some stage of growth.

Modern techniques may be useful in maintaining balance between over and under diagnosis of CRC at early stage. The contemporary molecular methods based on the large availability of high-throughput technologies are swiftly taking ground among other techniques. They ensure personalised approach by detecting patient-specific individual mutations, allowing for precise diagnosis, targeted therapy and evaluation of the future disease risk assessment. We have chosen and listed most promising methods to present them for your recognition.

The first technique is a single molecule, real-time, circular consensus sequencing (SMRT-CCS), that serves to detect mutations associated with CRC. The analyte is stool DNA containing exfoliated intestinal cells. The mutations utilised as markers in the test have been known for years: heterozygous mutations in APC (codon 1416, deletion 1c), KRAS (G13D caused by a transversion), and TP53 (Ser241Pro caused by transition) [1]. SMRT-CCS, alike the following techniques, is promising due to its specificity, sensitivity and progressive decrease in costs of sequencing analysis.

The next molecular method is targeted error correction sequencing (TEC-Seq) allowing for ultrasensitive direct evaluation of changes in circulating cell-free DNA using massively parallel sequencing. The approach, developed in 2017, uses COSMIC database as a background for targeting tumour-specific mutations [3]. Nowadays, we have dozens of genetic databases. Stored data keep expanding in the course of time and become invaluable source of information.

Another method using genomic database is the identification of CRC-restricted microRNAs, based on high-throughput data downloaded from the National Center of Biotechnology Information - Gene Expression Omnibus. MicroRNAs are known to be highly specific and are considered as a powerful tool in the state-of-the-art sequencing [4].

Different, but equally interesting approach, is high-throughput proteomics integrated with gene microarray. This method is yet in research phase, but out of already collected data we find the technique very promising. It consists of mRNA microarray integrated with mass spectrometry protein quantification. This accurate testing had been used for identifying potential biomarkers, nothing stands in the way thou, for using such precise method for diagnostic purposes [5]. Research gave over 200 potential gen-protein targets that may be useful in prophylaxis, even in light of everlasting discussion if biomarkers should or should not be used for screening. Testing consisted on method-by-method confirmation is the essence of personalised approach where every patient is viewed individually.

Parallel to advances in genetics we note evolution of other techniques. Chen et al. prepared an impressive catalogue of potential markers that could be utilized for diagnostic purposes. The list had been rolled on the basis of test sufficiency to detect an early stage cancer. In the search for potentially useful proteins, they found 64, among which four were considered the best due to their high specificity and adequate sensitivity: Anti-TP53, anti-IMPDH2, anti-MDM2 and anti-MAGEA4. These autoantibodies’ markers are recommended to be further tested in research on constructing new screening patterns [6]. The goal is to develop a balance in use of laboratory and imaging techniques. Properly conducted test should prevent patients from unnecesary colonoscopy and help to detect cancers that remained hidden despite endoscopic diagnostics. Clinical research is crucial for enrolling new tests in screening prophylaxis, that will answer the question about the usefulness of aforementioned methods and their impact on reducing CRC incidence as well as mortality, which is the prerequisite for effective screening.
